# Latest Developed Strategies to Minimize the Off-Target Effects in CRISPR-Cas-Mediated Genome Editing

**DOI:** 10.3390/cells9071608

**Published:** 2020-07-02

**Authors:** Muhammad Naeem, Saman Majeed, Mubasher Zahir Hoque, Irshad Ahmad

**Affiliations:** 1 Department of Life Sciences, King Fahd University of Petroleum and Minerals (KFUPM), Dhahran 31261, Saudi Arabia; g201908410@kfupm.edu.sa (M.N.); g201805240@kfupm.edu.sa (M.Z.H.); 2 Department of Chemistry and Biochemistry, Texas Tech University, Lubbock, TX 79409, USA; saman.majeed@ttu.edu

**Keywords:** CRISPR/Cas9, gene targeting, targeting specificity, homology dependent repair, base editors, non-homologous end joining repair pathway

## Abstract

Gene editing that makes target gene modification in the genome by deletion or addition has revolutionized the era of biomedicine. Clustered regularly interspaced short palindromic repeats (CRISPR)/Cas9 emerged as a substantial tool due to its simplicity in use, less cost and extraordinary efficiency than the conventional gene-editing tools, including zinc finger nucleases (ZFNs) and Transcription activator-like effector nucleases (TALENs). However, potential off-target activities are crucial shortcomings in the CRISPR system. Numerous types of approaches have been developed to reduce off-target effects. Here, we review several latest approaches to reduce the off-target effects, including biased or unbiased off-target detection, cytosine or adenine base editors, prime editing, dCas9, Cas9 paired nickase, ribonucleoprotein (RNP) delivery and truncated gRNAs. This review article provides extensive information to cautiously interpret off-target effects to assist the basic and clinical applications in biomedicine.

## 1. Introduction

Genome editing, a powerful toolset involving deliberate changes in the DNA sequence, is leading to a new era of molecular medicine against cancer, genetic disorders and beyond [1]. Editing methods currently available include zinc finger nucleases (ZFNs), transcription activator-like effector nucleases (TALENs) and clustered regularly interspaced short palindromic repeats (CRISPR). CRISPR was identified as the antiviral immune system of archaea and bacteria and later adopted as a competent tool for gene editing [2]. CRISPR/Cas9 is extensively used for gene editing than ZFNs and TALENs due to cost-effective, highly efficient, easy to use and highly effective [3]. The components of CRISPR/Cas9 are guide RNA (gRNA) and Cas9 nuclease. gRNA is a custom-designed short nucleotide base RNA that recruits Cas9 to cleave and edit specific loci in the genome. The CRISPR/Cas9 induce specific double-strand breaks (DSBs) inside double-strand DNA that trigger DNA repair pathways, such as non-homologous end joining (NHEJ) to introduce frameshift mutation for gene knock out (KO) or homologous direct repair pathway (HDR) for gene substitution or gene knock-in (KI) through supplied template DNA [4]. CRISPR/Cas9 genome editing has exploited in bacteria [5], plants [6] and mammals [7,8]. CRISPR/Cas9 has therapeutic potential in future clinical genetics, which leads to the approval of clinical trials in the USA to reduce genetic diseases [9]. Clinical trial data and comparative characteristics between CRISPR/Cas, ZFNs and TALENs on the nuclease platform depicted in Table 1.

**Figure 1:**
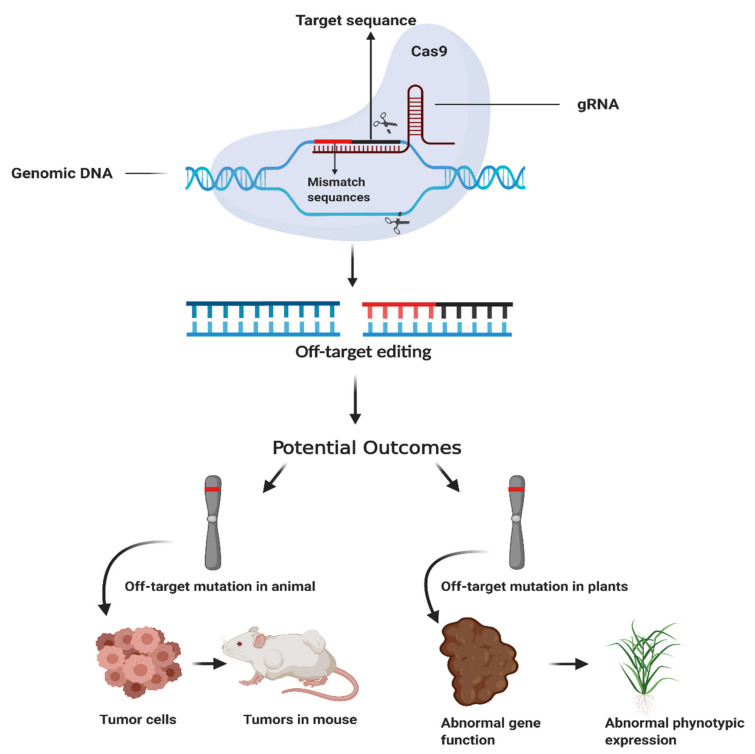
Effects of off-target mutation on animal and plant phenotype. Off-target causes genetic mutations. In clustered regularly interspaced short palindromic repeats (CRISPR)/Cas9 system gRNA sometimes binds other than target loci, off-target site. It may activate the oncogenes that initiate tumor cell formation in the animal body or may change gene function that leads to undesirable phenotypic expression (sensitive to diseases) in plants. The figure has been created with BioRender.com.

Several studies have revealed that Cas9 binds to unintended genomic sites for cleavage, termed as off-target effects [10]. The target efficiency of CRISPR/Cas9 determined through 20 nucleotide sequences of gRNA and PAM sites adjacent to target loci. More than three mismatches between target sequences and 20 nucleotides of gRNA can result in off-target effects [11]. It has demonstrated that 4 mismatches in PAM-distal end induce off-target effects [12]. Researchers have proposed two types of off-target effects, the first types of off-target effects likely to occur due to the sequence homology of the target loci and the next types of off-target sites occur in the genome other than the target site. Off-target effects cause a severe type of problem in the organism at the genomic level, large deletions and genomic rearrangements, which rarely could occur as a consequence of dsDNA breaks [13]. Off-target effects could lead to lethal genetic mutations that cause loss of gene function, ultimately cancer cells in animals and undesirable phenotype (disease sensitivity) in plants [14] (Figure 1).

The CRISPR–Cas9 system has the advantage that it can be transferred in distinct forms viz; Cas9–gRNA ribonucleoprotein (RNP), in plasmid/viral vectors and Cas9, mRNA with a distinct gRNA. In vivo delivery of CRISPR/Cas9 through viral vectors linked with some challenges for clinical applications, including; prolonged expression of CRISPR–Cas9, relatively high cost of production, immunogenicity and unintended mutagenesis in addition to editing in the off-target tissues [15]. Preclinical development for any genome editing treatment demands mitigation and thorough study of off-target risks before direct testing in humans. The selected loci is ideal if it has a relatively low degree of homology with the remaining genome sequence. This article reviews and highlights the CRISPR/Cas9 linked to the latest developed strategies to decrease the off-target effects or limitations in CRISPR mediated genome editing.

## 2. Mitigation of Off-Target Effects: Biased and Unbiased Off-Target Detection Methods

Native CRISPR/Cas9 system is an adaptive immune system in bacteria that protect the bacterial genome integrity from invading viruses [4]. The Cas9 specificity is very high in bacteria due to the small genome size than the eukaryotic genome, which is 1,000-fold larger than bacteria. Cas9 in bacteria has evolved without selection pressure; hence, there are high chances of off-target effects in the mammalian genome than bacteria [18]. Off-target cleavage sites have been detected in gene-editing techniques, CRISPR/Cas9, ZFNs and TALEN [19] but also in gene knockdown tools such as RNA interference (RNAi) and antisense [20,21]. Off-target effects in gene editing cause severe risks in clinical therapies. Researchers have discovered in vivo/vitro biochemical assays and algorithm-based computational approaches to detect and quantify the off-target effects to increase gene editing efficiency. Off-target detection methods categorized into two groups, biased and unbiased detection.

### 2.1. Biased

Careful designing of gRNA is crucial for the precise CRISPR/Cas9 targeting. For successful CRISPR/Cas9 research, off-target effects can be predicted through in silico tools [22]. The author identified that CRISPR/Cas9 could generate off-target sites under certain conditions; further, many scientist start to produce abundant data on off-target sites by using different CRISPR systems with different strategies [23,24,25]. Data generated is used to develop algorithmically based in silico predictive models to detect and quantify off-target effects. In silico prediction can minimize off-target effects through algorithmically designed software. Algorithmic based models were categorized into two groups (1) alignment-based models, Conventional algorithm based in which gRNA aligned to the reference genome and off-target effects returned on the base of sequence homology; (2) Scoring based models, advanced algorithmically designed, the most appropriate gRNA picked for the experiment from the given scores and ranking based on identified off-targets sites [26,27]. Biased methods illustrated in Table 2.

#### 2.1.1. Alignment Based Models

In this, the off-target sites predicted after aligning the gRNA sequences with reference genome sequences on a homology basis. It includes Bowtie, BWN, CasOT, Cas-OFFinder, FlashFry and CrisFlash. Bowtie and BWN are ideal tools to detect off-target effects based on simple alignment, which can detect off-target up to one mismatch [28,29]. The CRISPR/Cas9 target online predictor (CCTop, http://crispr.cos.uni-heidelberg.de), utilizes Bowtie to detect off-target activities that identify the PAM site to match and mismatch with protospacer sequence and then search by different parameters. In CCToP, up to five mismatches are allowed, ˃five mismatches prevent or inhibit the DSB creation [30]. CRISPOR, designing the gRNA tool, uses BWA to figure out off-target activities rather than Bowtie. CRSIPOR can predict all off-target effects [31]. Cas-OFFinder is a universal tool to detect off-target sites. The advantages of Cas-OFFinder are an unlimited number of PAM variety, mismatches and gRNA length. Off-target sites can figure out with Cas-OFFinder with only one base pair deletion/insertion [32]. CasOT, an algorithmically computational tool used to detect on-target sites from input data sequences, can spot all potential off-target sites ~6 mismatches (12-nt adjacent PAM) in the seed. This tool is useful to detect whether the off-targets are in exon or introns [33]. Recently, models viz., FlashFry and Crisflash were developed. FlashFry rapidly finds latent off-target sites and provides valuable information about GC contents, on/off-target score for targeted loci [34]. While Crisflash utilizes a tree-based algorithm for designing gRNAs and incorporates user-supplied variant data to increases its accuracy [35]. Among alignment-based methods, Cas-OFFinder is a better model for finding potential off-target sites, whereas FlashFry is efficient with its overall results and high in speed [27].

#### 2.1.2. Scoring-Based Models

It includes MIT, CFD, CRISTA, Elevation and DeepCRISPR. MIT (Hsu-Zhang) score was present to find out potential off-target sites in the course of the early stages of gene editing. Author evaluated > 700 gRNAs variants. Based on mismatch positions in target 20 bp, the weight matrix calculated to determine the off-target effects for each individual gRNA. MIT score incorporated in CHOPCHOP and CRISPOR sgRNA designing tools [31,41]. Cutting frequency determination (CFD) considered a prevalent score for off-target evaluation. Guide-seq used for CFD score validation, which performed better than the MIT score [36]. CFD score integrated with gRNA designing Tools viz., GuideScan and CRISPOR [42,43]. CRISPR target Assessment (CRISTA), which is an off-target search tool, has numerous features, including GC contents, RNA secondary structure, DNA methylation or epigenetic factors, to envisage cleavage efficiency. CRISTA performs well than CDF, CCTop and MIT [38].

Different studies have reported that DNA accessibility (DNA methylation, and chromatin structure) can affect the CRISPR cleavage in cells. To overcome this issue, the Doenche laboratory has developed two layers regression model, Elevation that takes both sequences and DNA accessibility/epigenetic data to find out potential off-target effects. Elevation can predict the individual off-target score for gRNA as well as the aggregate score for gRNAs. Elevation can perform better than MIT, CDF and CCTop. However, the only limitation is that it computes the off-target sites only for human exome (GRCh38) and does not work with different organisms [39]. Another latest developed tool, DeepCRISPR, is used in computational biology that predicts on-target and off-target cleavage sites simultaneously. It also includes epigenetic features affecting the KO/KI sgRNA efficiency [40]. Among the scoring based methods, the Elevation method proved to be the latest active and efficient tool to find out all potential off-target cleavage sites [36,44].

### 2.2. Unbiased

The unbiased methods can detect unintended cleavage sites in living cells on the whole genomic level. These methods categorized into in vitro detection or in vitro genome-wide assays and in vivo detection or cell-based genome-wide assays. Unbiased tools illustrated in Table 3.

#### 2.2.1. In Vitro Detection

In vitro genome-wide assays to detect and quantify the off-target effects mostly include Digenome–seq, SITE–seq and CIRCLE–seq. Digested genome sequencing (Digenome–seq) is a robust, sensitive and widely used method to detect genome-wide off-target effects of Cas9 and other nucleases. The sensitivity of Digenome–seq is 0.1% or lower. Digenome–seq requires high read depth, which makes it less sensitive and not suitable for screening large numbers of gRNAs [45,46]. Selective enrichment and identification of tagged genomic DNA ends by sequencing (SITE-Seq) and circularization for in vitro reporting of cleavage effects by sequencing (CIRCLE–seq) [2] are the most popular techniques developed to overcome the Digenome–seq issues. All the Cas9 cleavage sites within a genome could be mapped by the SITE-Seq method. In which performed sgRNA and Cas9 RNPs were used to cleave purified genomic DNA in a cell-free system. Next, both on- and off-target cleavage fragments are tagged, subjected to next-generation sequencing (NGS) to detect off-target sites. Nuclease concentration depends significantly on the total amount of the off-target sites. RNPs (low to high) used as variable concentrations for the recovery of low and high cleavage sensitivity off-target sites. Low concentrations of RNPs have a high propensity for off-target mutations when subjected to identification in the cells.

Additionally, SITE-Seq requires minimal NGS read depth than Digenome–seq [47,48]. CIRCLE-Seq has a similar concept with some procedural differences. In CIRCLE-Seq, the DNA initially trimmed, circularized, ultimately residual DNA is degraded. The degradation step, before treatment with (Cas9–sgRNA) RNPs, virtually eliminates high background DNA to increase sensitivity, which condenses NGS read space, wasted on random reads [49]. Next, DNA is linearized by cleavage with Cas9 and further subjected to NGS for off-target detection. Just as in SITE-Seq, CIRCLE-Seq does not require a reference genome, and additionally, it can also detect off-target sites that are diminished or enhanced as a result of cell-type-specific single nucleotide polymorphisms (SNPs). However, the circularization step requires a large quantity of genomic DNA, which could be a limiting factor for this method [47]. The number of cleavage sites spotted with SITE-Seq and CIRCLE-Seq is higher than that detected in live cells during cell-based genome-wide assay detection, because of the lack of associated proteins besides the higher-order chromatin structure or epigenetic changes [47]. Compared to Digenome–seq and SITE–seq, CIRCLE–seq is better as an in vitro genome-wide assay that can detect 94% off-target sites. However, CRISLE-seq can work in specific cells that labeled with the GUIDE–seq (cell-based in vivo off-target detection assay) tag [47].

#### 2.2.2. In Vivo Detection

In vitro and cell-based cleavage, condition differs significantly, many off-target determined by In-vitro are not cleaved (repaired) in vivo; therefore, there was a need to develop in vivo or cell-based genome-wide assays to detect real off-target effects. The widely used cell-based genome-wide assays to detect off-target sites includes, integrase defective lentiviral vectors (IDLVs), chromatin immunoprecipitation and high throughput sequencing (Chip–seq), breaks labeling, enrichments on streptavidin and next-generation sequencing (BLESS), genome-wide, unbiased identification of DSBs enabled by sequencing (GUIDE–seq), Discovery of in situ Cas off-targets and verification by sequencing (DISCOVER–seq), genome-wide off-target analysis by two cell embryo injection (GOTI) and verification of in vivo off-targets (VIVO). IDLVs is the earliest tool to identify genome-wide off-target sites of ZFNs. IDLVs is transferred simultaneously with the gene-editing system, which integrates with DSBs. The DNA extracted and sheared with suitable size and fragments are ligated through a linker. Ultimately PCR was performed to check IDLVs distribution to find out off-target effects [58]. IDVLs is effective off-target detecting method in those cells which are difficult to transfect like primary human cells. IDLVs demerits include, it cannot analyze multiple off-target sites and its off-target detecting efficiency is 1% [51]. Chip–seq is an indirect in vivo off-target detecting method on protein DNA binding basis [59]. In this tool, Cas9 replaced with dCas9. It can bind to DNA without DSBs, which enable off-target detection sites [60]. However, the dCas9-gRNA complex in Chip–seq can alter the specificity of off-target detection [60,61]. BLESS is the latest method for the genome-wide mapping of DSBs introduced by Cpf1, Cas9 and nucleases. This technique offers the advantage of being more versatile, sensitive, and quantitative than other DSB mapping methodologies. Additionally, the researchers demonstrated that this technique is also applicable to exogenous and endogenous DSB detection in low input cells and tissue samples [53]. It applies to mouse and human cell lines. However, the limitation of BLESS includes, it only detects off-target effects during the labeling period and reference genome is required. GUIDE–seq based on the incorporation of oligodeoxynucleotides (dsODN) in DSBs followed by the NHEJ DNA repair pathway and the integrated dsODN later amplified to detect and quantify the off-target effects [54]. It has limitation like the requirement of high-level sequencing read depth to eliminate the problem of high background. However, it reported that GUIDE-Seq is capable of detecting the off-target sites that are not identified by ChIP-seq [62]. Additionally, it can detect the RNA-guided-nuclease independent DSB hotspots within the genome [26,63], the efficiency of GUIDE–seq limited by Chromatin accessibility or epigenetic factors [54].

LAM–HTGTS is a robust, sensitive and unbiased in vivo off-target detection method [64] which can detect the chromosomal translocations in cultured mammalian cells, due to end joining of endonuclease induced DSBs. This method depends on the joining of “bait” DSBs and “prey” DSBs due to which translocations may occur. Genomic DNA is extracted, sheared and the bait-prey junctions amplified by LAM-PCR and prepared for NGS analysis. The advantages of LAM–HTGTS include the ability of the method to identify all known classes of genome-wide recurrent DSBs induced by on/off-target activity of engineered nucleases. LAM–HTGTS only identify the specific DSBs that translocate, which is considered a loophole in this method as translocation events due to DSBs rarely compared to rejoining events that witnessed as local deletions and insertions [26,64]. The LAM–HTGTS applies to the human genome and its efficiency limited by chromatin accessibility or epigenetic factors [65]. DISCOVER-Seq is a dominant, sensitive cell-based genome-wide assay for in vivo off-target detection [55]. MRE11, ChIP-Seq and custom web tool BLENDER (blunt end finder, https://github.com/staciawyman/blender) are the main attributes of DISCOVER-Seq. The MRE11, a DNA repair factor, is recruited to DSBs by endogenous DNA repair mechanisms. Then ChIP-Seq is utilized for the precise analysis of the MRE11 sites over the genome for off-target site identification. One advantage of DISCOVER-Seq over GUIDE-Seq is that the former technique prevents the cytotoxic effects induced due to the transfer of exogenous tag dsODNs in GUIDE-Seq technique. It is applicable to human and mouse cell lines. This enables the off-target site detection in human induced pluripotent stem cells (hiPSCs) [26,55]. The latest developed tools to detect unbiased in vivo off-target effects are GOTI and VIVO. GOTI latest developed to evaluate the off-target effects occurs through Cas9, cytosine base editors 3 (CBE3) and adenine base editors -7.10 (ABE 7.10) [56,66]. GOTI detects the off-target sites in the cell population resulting from single gene-edited blastomere. It can detect off-target sites in the mouse embryo at the early stages. VIVO is the latest tool to detect off-target caused due to CRISPR/Cas9. VIVO demonstrated that correctly designed gRNA could edit the mouse genome efficiently with minimal off-target sites. Comparable to all cell-based assays, GUIDE–seq preferred due to less costly, low numbers of components, low false-positive rates, applicable to diverse cell lines, and can detect the low abundance of off-target sites [67].

## 3. Mitigation of Off-Target Effects: Guide RNA (gRNA) Modification and Engineering

The gene-editing by CRISPR/Cas9 mainly depends on gRNA specificity (how much gRNA bound to target site) and efficiency (how much gRNAs create DSBs at the target site), which helps in the cleavage of the genome by guiding Cas9; however, to design an efficient gRNA with low off-target effects is a challenging task [68]. The applicability of CRISPR/Cas9 in a broader perspective depends on its capacity to target DNA based on a synthetic sgRNA sequence, i.e., twenty (20) nucleotides guide sequence occupying at the 5′ end of the gRNA sequence. It has reported that the gRNA sequence is considered a key factor to be reflected in enhancing cleavage efficiency and targeting specificity [36]. In contrast to conventional genome editing tools viz., ZFNs, TALENs, and CRISPR/Cas9 technique have an advantage due to its multiplex targeting mutations that can be created by the real-time introduction of many gRNAs into the cells [69]. Additionally, perfect gRNA can be used for the minimum off-target and maximum on-target mutagenesis. CRISPR/Cas9 system is widely applicable to enhance or reduce the expression of a targeted gene, knock-in and knock-out genes [70]. Hence, it is necessary to carefully design, engineer and modify the gRNAs to circumvent off-target mutations. Different strategies, such as GC contents, gRNA length, truncated gRNA and chemical modification have been developed to reduce off-target effects.

### 3.1. GC Content of sgRNA

Researchers discovered that the structural sequence of gRNA has an impact on the on-target activity of CRISPR/Cas9 gene editing. Structural analysis of gRNA performed to test its activity [71]. The GC contents between 40%–60% in the gRNA sequence increase the on-target activity [72] because higher GC content stabilizes DNA: RNA duplex and destabilize the off-target binding. The position of gRNA-on-gRNA sequence effects on editing, purine residue position at the end of four nucleotides increase editing efficiency. The guanine preferred at 20 position of gRNA and Cytosine at 16 positions to increase on-target editing [71,73]. Consistent with these findings, a positive correlation among PAM-proximal part GC percentage of the sgRNAs through mutagenesis has been investigated [74]. That parameters help to design better gRNA.

### 3.2. Length and Mismatches

Many studies revealed that unwanted mutations could be affected by the length of gRNA, such as its length up to 17 nucleotides revealed a higher genome-editing efficiency. In contrast, its length (18–20 bp) revealed low genome editing efficiency [75]. Moreover, 20 bp gRNA not observed to linked with any unwanted mutations [76]. Additionally, partial DNA replacement strategy, along with chimeric guide RNA exploited to decrease off-target effects in primary human cells [77]. The guidelines to tail off the potential off-target effects were reported in human cells viz; 1) target sequence has greater than three mismatches within the range of 7–10 bp of the PAM should be avoided and 2) inside 12 bp of the PAM, sgRNA bulges should be avoided to reduce off-target effects [78].

### 3.3. Truncated gRNA

Off-target events reported decreased by 500-fold, without affecting on target accuracy, by shortening the gRNA length over the first 20 bp to 17/18 bp [75,79]. On the contrary, unintended minimum changes were observed in mammalian cells when 17 bp sgRNA was used [75]. The three nucleotides at 5′ end of gRNA significantly decreased undesirable effects in mammalian cells system. Combining the gRNA and Cas9 with a paired nickase significantly decreased the off-target effects in mammals [79]. Dead RNA off-target suppression (dOTS) is the latest developed strategy in which dead truncated gRNA that direct Cas9 binding, but suppress cleavage, has resulted in decreased off-target effects and increase on-target activity 40-fold [80].

### 3.4. Chemical Modification of gRNA

Incorporation of 2ʹ-O-methyl-3ʹ- phosphonoacetate in the gRNA ribose-phosphate backbone leads to site-specific modification causing 40–120-fold reductions in off-target cleavage while preserving on-target performance [81]. Specificity improved ~25,000-fold at off-targets by the integration of bridged and locked nucleic acids in the guide sequences by the formation of a dynamic RNA-DNA duplex [82]. Long term expression of the CRISPR system causes many off-target effects [77]. Designing and delivering a self-restricted CRISPR system by co-expressing gRNA to target Cas9 that restricts the expression of CRISPR leads to a reduction in off-target effects [83]. Modification of hairpin structure at 5′ upstream of gRNA improves the specificity of Cas9 and Cas12 ~55-fold decreasing the off-target effects [84].

## 4. Mitigation of Off-Target Effects: Improved Cas Variants

*Streptococcus pyogenes* Cas9 (SpCas9), which is the more broadly utilized Cas9, generates genome-wide off-target mutations, which led scientists to develop variant SpCas9 s and other Cas9 orthologous that can solve the issue to some extent. Here we have classified Cas variants on the base of their origin. The Cas nuclease variants illustrated in Table 4.

### 4.1. SpCas9 and SaCas9 Variants

#### 4.1.1. SaCas9, SpCas9-Nickase, dCas9 and dCas9–FokI with Multiple Fusion Domains

**Figure 2:**
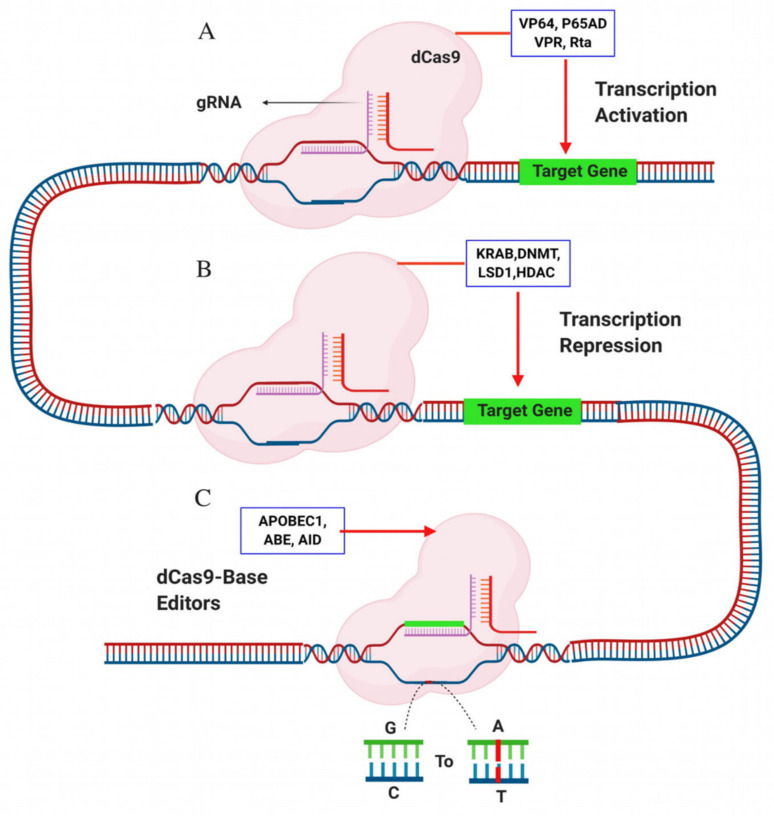
Deactivated Cas9 (dCas9) used for gene activation, repression and base editing. (A) Activations domain such as VP64, P65AD, VPR, Rta fuse to dCas9 to re-activate the gene expression; (B) repression domains such as KRAB, DNMT, LSD1 and HDAC fuse to dCas9 to repress/decrease the gene expression; (C) APOBEC1 (cytidine deaminase) and ABE (adenosine deaminase) fuse to dCas9 for efficient base editing. The figure has been created with BioRender.com.

Adeno-associated-virus (AAV) is an appropriate vector for ex vivo or in vivo gene therapy due to its low immune response and ability to infect broad cells. Packaging of SpCas9 in AAV is restricted due to large size > 1 kb. To overcome this issue, SaCas9, a small size < 1 kb Cas9 variants derived from *Staphylococcus aureus,* can easily be transferred through the AAV vector. SaCas9 also recognizes a more extended PAM sequence (5ʹ-NNGRRT-3ʹ) than the much shorter 5ʹ-NGG-3ʹ sequence recognized by SpCas9. In theory, this means that SaCas9 expected to recognize its PAM sequence once every 32 bps of a random DNA (SpCas9 would recognize its PAM every eight bps). It indicates that genome editing with SaCas9 may have reduced the chances of off-target mutations [98]. It reported that genome editing was successful with SaCas9 in RNP form in human cell lines, including the correction of a pathogenic *RS1* mutation for X-linked Juvenile Retinoschisis. Targeted deep sequencing did not reveal any off-target effects [99]. Paired Cas9 nickase is the mutated form of SpCas9 generated through mutation of the HNH or RuvC domain. Cas9 nickase creates a nick in one strand, so there is a need for a pair of gRNAs to make an efficient double-strand break. In turn, this will increase the specificity of the action [65]. Dead Cas9 (dCas9) is also another mutated variant of SpCas9; both HNH and RuvC domains are mutated. dCas9+gRNA serves only the specific DNA-targeting, which fuses to transcription activation, repression and base editing domains to create epigenetic (gene activation or repression) and base changes in the genome rather than creating DSBs (Figure 2).

The transcription activators domains such as VP64, P65AD, VPR, Rta and TETI are fused to dCas9 to increase gene expression [100,101]. Further to increase the gene expression different groups have developed three types of systems, VPR (triplet combination of VP65-p65-Rta) [102], synergic activation mediator (SAM, dCas9-V64 fused with gRNA 2.0 and transcription helpers MS2-p65-HSF1) [102] and Sun tag activation system (V64 bound with CGN antibody) [103]. SAM activation system has more potent to reactivate gene expression than VPR [104]. A group reactivated the latent HIV gene with fewer off-target effects in microglial cells [104]. A group activated CXCR4 gene expression effectively in K562 cell lines with a sun tag activation system [103]. RNA guided *F*okI nuclease (RFNs) or dCas9–FokI is a Cas9 variant formed by the fusion of dead Cas9 (dCas9) and *F*okI nuclease domain. For transcription repression, multiple domains such as KRAB [103], DNMTs and LSD1 fused with dCs9 to decrease the gene expression [105]. In comparison to other repressor domains, KRAB is most efficient in gene silencing or repression [106]. With dCas9 fused with repression domains (KRAB, DNMT, LSD1, HDAC) can efficiently decrease the expression of gene up to 99% than earlier RNA interference (RNAi) technology [107]. The specificity of dCas9–FokI is 140 times higher than wild type SpCas9 in human primary cells [108]. Recently, a group developed engineered chimeric RFNs/dCas9–FokI variants (GGGGS, GGGGS_5_, EAAAK_5,_ XP_3_). dCas9–FokI variant, GGGGS_5_ outperformed with no detectable off-target effects in HEK293 T cells [109]. Base editors are a new irreversible approach to edit the DNA basis includes cytosine base editor (CBE) with cytidine deaminase that convert C–T and adenine base editors (ABE) with adenosine deaminase that convert A–G. The fusion of dCas9 with cytidine deaminase enzyme, APOBEC1 (dCas9-cytidine deaminase) or adenosine deaminase (dCas-adenosine deaminase) is another alternative approach to enhance the efficiency of base editors with low off-target effects [110,111]. Edition of specific loci, a potential treatment of genetic disasters and crop trait enhancements could be achieved using both Cas9 nickase and dCas9 [85,112].

#### 4.1.2. xCas9, Cas9-NG, evoCas9

Off-target effects minimized and specificity increased by engineered variants of spCas9, xCas9 and Cas9-NG. The variant of xCas9 xCas9-3.7 efficiently targets ~16 NGN PAMs, combinations like GAA, NG, NGG and GAT. In this regard, GAT, GAA and NG are some examples of a broad range of PAMs recognized by xCas9. Compared to SpCas9, xCas9 has a higher specificity and low unintended genetic modification in human cells [113]. Cas9-NG recognized as the relaxed PAMs for plant gene editing. It has higher specificity in the plant, but in animals introduces indels with NG PAMs site [114]. A direct evolution of SpCas9 and random mutation in its REC3 domain results in the discovery of evoCas9 mutants. GUIDE–seq was used to assess the efficiency of evoCas9 mutants at eight different loci. The on-target activity was significantly higher than off-target activity in evoCas9 than wild type SpCas9 [89].

#### 4.1.3. SpCas9–HFI, eSpCas9, Hypa-Cas9

Engineered High-Fidelity SpCas9 variant, SpCas9-HF1 has shown exceptional accuracy with ˃85% of the sgRNAs confirmed in human cells while exhibiting negligible off-target effects. It is similar to WT SpCas9 due to its extraordinary performance and could potentially use in therapeutic applications [115]. For human cell genome editing, enhanced specificity SpCas9 (eSpCas9) considered a useful tool. A rationally generated eSpCas9 (K848A, K1003A, R1060A) can reduce non-target strand binding and therefore enhance the specificity of gene editing [116]. However, both SpCas9–HFI and eSpCas9 are poorly active at some target loci at the genomic level [117,118]. To overcome the poorly active status of SpCas9–HFI and eSpCas9 at many loci, hyper-accurate Cas9 engineered through point mutation in the REC3 domain of SpCas9. Hyper-accurate Cas9 Variant (Hypa-Cas9) show higher on-target activity in human cells without off-target effects [86].

#### 4.1.4. Hypa-Cas9, Sniper–Cas9, HiFi Cas9, SpG and PAM-less SpRY

Another Cas9 variant, Sniper–Cas9, developed through direct evolution in E.coli bacteria. With truncated gRNA and RNP delivery, Sniper–Cas9 specificity is significantly higher than SpCas9–HFI, eSpCas9 and evoCas9 mutants in human cell lines [87]. Recently, highly versatile Cas9 variant, HiFi Cas9, is developed for the on-target activity in the primary human cells. As compared to all other Cas9 variants, Hifi Cas9 is shown slightly low on-target activity, but is highly versatile and more active on many loci [119]. SpG and SpRY are latest developed Cas9 variants, SpG can target expanded set of NGN PAMs and SpRY is the latest developed nuclease variant that used to target all PAM sites in human cells effectively [120]. Engineered SpRY paved a way to develop new gene-targeting techniques with PAM flexibility.

### 4.2. Improved Cas9 Orthologous with Broader PAM Capability and Specificity

#### Cpf1, CRISPR-Cpf1-RNP

Another RNA guided endonuclease initially identified in *Prevotella* and *Francisella* 1 is Cpf1 (or Cas12a). CRISPR/Cas9 and CRISPR/Cpf1 systems have some differences between them. Cpf1 recognizes 5′-TTTV-3′ PAM and requires the only crRNA (without tracrRNA) for functioning [121]. According to the study, CRISPR/Cpf1 is less efficient (0%–60% mutated T0 plants) than CRISPR/Cas9 (90%–100% mutated T0 plants) in generating on-target mutations when used for genome editing in maize. Additionally, their results also suggest that Cas9 is comparatively specific as sequencing did not detect any mutations at off-target loci in T1 plants that were constitutively expressing Cas9 and gRNAs [114]. Recombinant CRISPR-Cpf1 Ribonucleoprotein (CRISPR-Cpf1-RNP) decreased off-target activity in mouse cells [122].

### 4.3. St1Cas9, NmCas9, FnCas9, CjCas9

Cas9 Orthologous St1Cas9, St3Cas9 derived from *S. thermophilus* that recognize the PAM sequence, 5′-NNAGAAW-3′, 5′-NGGNG-3′, respectively. St1Cas9 show high on-target activity than WT SpCas9 [94,123]. NmCas9 derived from *N. meningitidis* that recognize the 5-NNNNGHTT-3′ PAM site, show high specificity, but low off-target site than SpCas9 [124]. FnCas9 derived from *F. novicida* that recognizes the 5′-NGG-3′ PAM site. FnCas9 can target DNA as well as RNA. The on-target activity is low than SpCas9 [125]. Recently, a new smallest Cas9 orthologous CjCas9 discovered, it has high on-target activity than SpCas9. CjCas9 is small in size than saCas9. It can be transferred to the target cell easily with the AAV delivery method. Due to its small size, CjCas9 has future promises in therapeutic gene editing [97].

## 5. Mitigation of Off-Target Effects: CRISPR Delivery Methods

The delivery methods used to introduce the sgRNA/Cas9 editing machinery has a role in the ultimate accuracy of the system.

### 5.1. Improved Viral CRISPR Delivery Methods

Adeno-associated virus (AAV), an example of viral vector delivery systems, has been extensively used to deliver gene-editing components in gene therapy. This virus offers an advantage of the negligible immune response (innate or adaptive) and is not known to associate with or cause human diseases [126]. However, it has drawbacks when used for delivering CRISPR/Cas9 gene-editing system Viral based delivery methods usually mean that the Cas9 and gRNA packaged into plasmid DNA which delivered via the viral vector to the target cell. As mentioned earlier, this allows the CRISPR/Cas9 components coding genes to exist persistently in the target cell type, which results in elevated Cas9 levels and increased chances of off-target activity [127]. Adeno viruses (AdV) naturally show meager potential to integrate into the target cell genome, a characteristic that is advantageous for limiting off-target effects. However, AdVs elicit immune responses and currently, it is not possible to completely rule out its integration into the host genome [128].

### 5.2. Improved Non-Viral CRISPR Delivery Methods

Overexpression of the editing components may increase off-target mutations when the editing machinery persists for a long time in the nucleus, observed during plasmid-based co-expression of Cas9 and sgRNA into the target cell [127]. However, if the Cas9 protein and gRNAs delivered as an RNP complex, the editing time window can be shortened, which reduces the off-target effects. Since the RNP form of the nuclease is free of exogenous DNA, it is quickly degraded in the cell or diluted in mitotic cell divisions [63]. Generally, the delivery methods can be grouped into either being viral or non-viral. Electroporation is a physical transfection method that uses pulse high voltage electrical currents as a result of which small pores are transiently formed on cell membranes. However, this method may not always be suitable for in vivo applications due to the high voltage applied across cell membranes that causes cell toxicity [129], though electroporation is not possible for in vivo CRISPR/Cas9 application. Electroporation showed low numbers of off-target mutations in plant protoplasts when the sgRNA and Cas9 delivered as RNP complexes [130]. Delivering RNP complexes by liposome-mediated transfection revealed the minimization of off-target mutations compared to plasmid DNA transfection [90].

Lipid nanoparticles are a ubiquitous delivery vehicle to transfer different molecules to target cells. It generally used for nucleic acid delivery that loaded inside cationic liposomes, which can easily pass through cell membranes of target cells. CRISPR/Cas9 components delivered as RNP complexes to achieve more success since Cas9 and sgRNA stay together due to their anionic properties [131,132]. Another delivery methodology uses vesicles (cell-derived nanovesicles) to deliver Cas9 and sgRNA as RNPs to target cells. Since RNPs, there is no risk of sustained expression of Cas9 (which elevates Cas9 concentration and escalates the risk of off-target editing) [133]. The effect of different transformation methods on the off-target mutations in plants has been reviewed [134]. The latest developed non-viral gene delivery method, polyethyleneimine (PEI) magnetic nanoparticles (MNPs) using biomaterials proved useful, nontoxic with low off-target effects, MNPs considered as the best strategy for CRSIR-Cas mediating gene-editing system [135]. Recently, researchers discovered that the co-transfection of the small vector (3 kb) and large vector (15 kb) into human cancer cell lines through electroporation or liposomal transfection methods have increased transfection efficiency by 40% and decreased the cell death by 45% [136].

## 6. Mitigation Off-Target Effects: Base Editors

Usually, unintended mutations or off-target effects occurs in the genome due to DSBs preceded by NHEJ for gene knock out or HDR for knock-In. The most powerful tool could be genome editing without DSBs. Recently, a new genome-editing technique has been developed for base editing, which can change specific nucleotides in the genome without introducing DSBs [111,137,138]. The base editing technique comprises dCas9, deaminase (catalytical base modification enzyme) and sgRNA. Compared to HDR, these base editing tools have shown significant gene editing efficiency. Base editors are more efficient in editing DNA of quiescent cells than Cas nucleases, which require endogenous DNA repair mechanism that is inefficient in non-dividing cells [66]. Cytosine base editors (CBE) and Thymine base editors are the two categories of base editors developed, which can change C/G to T/A and A/T to G/C, correspondingly.

### 6.1. Cytosine Base Editors (CBEs)

Cas nucleases, sgRNA and AID/APOBEC cytidine deaminases are the main components of CBEs. In DNA, CBEs generate thymine base (T) by hydrolytic deamination of Cytosine (C) catalyzed by the two enzymes APOBECs (Apolipoprotein B mRNA Editing Catalytic Polypeptide-like) and AID (activation-induced cytidine deaminase) [139]. The first generation of CBE (BE1) is a fusion of rat APOBEC with dCas, which is prone to inactivation by mutations of D10A and H840A and able to convert Cytosine to thymine (between 12–16 range from PAM site of sgRNA). The second-generation of CBE (BE2) is formed by adding uracil-DNA glycosylase inhibitor (UGI) to BE1 that increases the cytosine base efficacy by three-fold [140]. To further increase the editing efficiency of base editor, third-generation base editor (BE3) was developed by combining Cas9n (inactivated by mutation D10A to create a nick in target strand), cytidine deaminase and UGI, which possess six-fold editing efficiency than BE2. As BE3 comparatively causes fewer off-target effects, it has gained extensive usage in animal (mice), bacteria and plant cells to edit the genetic makeup of the cell. For better inhibition of endogenous base excision repair, another UGI copy and BE3 fused to develop fourth-generation base editor (BE4). Editing efficiency exhibited by BE4 is superior compared to BE3. Another critical factor is DNA methylation that inhibits the base editing efficiency. The human hA3A-derived BE system (hA3A–BE3) developed for the base editor, which shows maximum editing efficiency in highly methylated DNA regions [141].

### 6.2. Adenine Base Editors (ABEs)

Adenine base editors (ABEs) created for changing Adenosine (A) to Guanosine (G). A series of ABE was developed based on *E. coli* TadA (ecTadA) enzymes. ABE 7.1 to 7.10 engineered to increase on-target activity with low off-target effects [111]. ABE-max produced through nuclear localization decreased off-target activities [142]. Recently, a newly developed base editor, ABE8e, increases 590-fold on-target activity than ABE 7.10 [143].

### 6.3. DNA & RNA Off-Target Mutations

Recently many off-target mutations throughout the genome have detected in rice and mouse embryo with an average frequency of 5.3×10^−7^ per bp and 5×10^−8^ per bp, respectively resulting from BE3, an original CBE [56,144]. The overexpression BE3 and ABEs induce off-target RNA mutations, which are sgRNA independent, *e.g.,* off-target caused by rAPOBEC1 and TadA-TadA* [56,145]. Optimization of rAPOBEC1 and TadA-TadA* is needed to reduce unintended mutations. The mutations (R33A or R33A/K34A) introduced in rAPOBEC1, which known as SECURE (S Elective Curbing of Unwanted RNA Editing), which shows a substantial reduction in RNA off-target activity [145]. The ecTadA or ecTadA* through point mutation (E59A) was inactivated, which has decreased RNA off-target effects. To increase further specificity, ecTadA* was improved by changing the specific three residues, which reduces unintended RNA off-target efficiency [146]. Recently, a single gene modification (D53E or F148A) was introduced in ecTadA to develop ecTadA* (mutant of ecTadA) that significantly decreases RNA off-targets [147].

### 6.4. Efficient Base Editors Delivery

One limitation of the base editor is the in vivo delivery to target tissues—ribonucleoprotein (RNP) delivery of base editor more efficient than plasmid delivery system comparatively. Hence, different delivery strategies ought to considered cautiously to decrease un-intended editing by controlling the exposure time [148]. As mentioned earlier, the AAV delivery vector is a popular method for *in-vivo* gene therapy [149]. Packaging capacity limits the delivery of AAV and the size of base editors (5.2 kb) precludes the packaging in AAV with Cas nucleases. Recently, a *trans*-splicing interns approach was developed for in vivo delivery of CBEs and ABEs, which achieve efficient base editing in skeletal muscle (9%), heart (20%), retina (38%), liver (38%) an mouse brain (~59% of unsorted cortical tissue) [150].

### 6.5. Broader PAM Sites Base Editors

To broaden the PAM recognition site, many research groups have replaced the spCas9 from BE3 with various engineered nucleases such as KKH SaCas9, EQR, VRER, SaCas9, VQR, xCas9 and SpCas9-NG, which recognize the different PAM sites in the genome [113,114,151,152]. Many types of ABEs developed through substituting the nCas9 with nXCas9 in ABE7.10, xABE that extended the genome target region containing GAT, GAA and NG PAM sites. All mentioned base editors target to recognize G/C rich PAM site. “CRISPR-Cpf1-based BE” was developed to overcome this limitation by fusing the rat APOBEC1 with non-catalytical Cpf1 nuclease enzyme, which recognizes the T-rich PAM site with relatively few un-intended modifications in human cells [153].

## 7. Mitigation of Off-Target Effects: Prime Editing

Recently, a new gene-editing method (Prime editing) developed by combining reverse transcriptase programmed with prime editing gRNA (pegRNA) and Cas-nickase nuclease that can edit or “search and replace” bases in mammalian cells without a double-strand break and DNA donor template with less collateral damage. The limitations of BEs covered by Prime Editing (PE), BEs can install efficiently four transition mutations (C to T or G to A, A to G and T to C). However, Prime editing can install all 12 possible transition alterations (C/A, C/G, G/C, G/T, A/C, A/T, T/A and T/G) in the genome. BEs require the target site to edit in a particular position to PAM. That is why unwanted bystander mutations occur with the occurrence of many Cytosine or Adenine bases inside the ‘’edit window’’ or lack of PAM position from 15 ± 2 relative to target edit. Whereas PE can edit when bystander edit is unacceptable or the target site lacks positioned PAM. The mutations have been corrected, which cause, sickle cell anemia and Tay-Sachs disease with minimal off-target editing and PE can correct up to 89% known, causing pathogenic mutations [154]. Prime editing is a premature technology. NHEJ could repair some of the sites; this could be considered as additional type off-targets. However, there is a need for more research on prime editing on animal and plant models to move it into therapeutic gene editing.

## 8. Mitigation of Off-Target Effects: Anti-CRISPR Proteins

Anti CRISPR (Acr) proteins are natural CRISPR/Cas system inhibitors which are encoded by various mobile genetic elements (MGEs), that inhibit the CRISPR-Cas immune function at various stages. Up to 45 Acr proteins have discovered from which “AcrIIA4” has the potential to protect the cell from editing [154]. Shin with coworkers discovered that AcrIIA4 decreases off-target modifications by four times without reducing on-target effects by adjusting the time when AcrIIA4 or Cas9 added to assay [155].

## 9. Conclusion and Future Outlook

The success of CRISPR/Cas9 to modify genome with unprecedented precision can be owed to its accuracy, efficiency, cost-effectiveness and ease of use than the classical gene-editing tools (ZFNs and TALEN). However, CRISPR/Cas tool causes deleterious off-target effects at the genomic level. Various techniques reported, including different bioinformatics approaches such as for in silico detection of off-target mutations along with improved on-target efficiency to ameliorate the off-target effects. Choosing an appropriate off-target detection tool, such as biased and unbiased with predictive on-target and off-target sites, is more critical than the CRISPR delivery system. Most preferred off-target detecting biased and unbiased methods are Elevation and GUIDE–seq, respectively. In gRNA modification, truncated sgRNA stipulates a simple way to reduce off-target effects and RNP based delivery is suitable in most cases to get higher on-target activity. Additionally, choosing Cas variants is also critical to reduce the off-target effects that depend on the nature of the experiment.

Additionally, choosing Cas variants is also critical to reduce off-target effects. Researchers are in the race of creating engineered Cas9 variants and new gene-targeting techniques with negligible off-target effects in mammalian cells for the improvement of therapeutic gene editing or genome surgery. The currently developed prime editing tool has future promises to treat genetic diseases with minimum off-target effects in human cells, but off-target effects also limit Prime editing applications. However, further study needs for the development of a super CRISPR system to treat genetic diseases in the healthcare system.

## Figures and Tables

**Table 1:** Comparison between ZFNs, TALENs and CRISPR/Cas on the nuclease platform and clinical trial data [16].

	Characteristics	ZFNs	TALENs	CRISPR/Cas
**Nuclease Platform**	Full-form	Zinc finger nucleases	Transcription activator-like effector nucleases	Clustered regularly interspaced short palindromic repeats
	Source	Bacteria, eukaryotes	Bacteria (*Xanthomonas* sp.)	Bacteria (*Streptococcus* sp.)
	Type of recognition	Protein-DNA	Protein-DNA	RNA-DNA
	Double-stranded break pattern	Staggered cut (4–5 nt, 5′ overhang)	Staggered cut (heterogenous overhangs)	SpCas9 generates blunt ends; Cpf1 generates Staggered cut (5′ overhang)
	Improved/other versions	AZP-SNase	Tev-mTALEN	Cpf1, eSpCas9
	Specificity	Low–moderate	Moderate	Low–moderate
	Cost (USD)	5–10,000	< 1000	< 100
	Efficiency/inefficiency	The small size of ZFNexpression cassettes allowuse in a variety of viralvectors	Packing intoviral vectors are difficult due to the large size of TALEN	Commonly used Cas9 from *S. pyogenes* is large, impose packaging problems in viral vectors
**Clinical Trials Data**	Pathology understudy	Hemophilia B, Transfusion Dependent beta-thalassemia, sickle cell disease, human papillomavirus-related malignant neoplasm, HIV	Human papillomavirus-related malignant neoplasm	Human papillomavirus-related malignant neoplasm, multiple myeloma, infections (HIV and gastrointestinal), sickle cell disease, thalassemia
	Cost	+++	++	+
	Recognition	Protein-DNA	Protein-DNA	RNA-DNA
	Region/No. of studies	East Asia/1, North America/13	East Asia/2, North America/3	East Asia/11, Europe/2, North America/8
	Status of studies	Out of 14 studies, 5 completed, 3 are currently recruiting patients, whereas 4 are active	Out of 6 studies, 3 are currently recruiting patients, whereas 3 carry unknown status and 1 withdrawn	Out of 21 studies, 15 are currently recruiting patients, whereas 1 is active, but not yet recruiting and 1 withdrawn

Nuclease Platform [17] Clinical Trials Data* (https://clinicaltrials.gov/ct2/home).Off-target effects severely obstruct the reliability as well as the accuracy of the CRISPR system. The symbol (+, ++ and +++) means low, moderate, and high cost respectively.

**Table 2:** Biased tools to detect and evaluate the guide RNA (gRNA) efficiency.

Tool	Description	Method	Web Source	Reference
CasOT	A biased genome-wide off-target detecting online tool required paired gRNA with unlimited mismatch number.	Alignment	http://eendb.zfgenetics.org/casot/	[33]
Cas-OFFinder	Very fast and versatile in silico based tool, detect off-target site with unlimited mismatch numbers.	Alignment	http://www.rgenome.net/cas-offinder	[32]
FlashFry	A fast tool to find off-target sites and provides valuable information about GC contents, on/off-target score for targeted loci.	Alignment	http://aaronmck.github.io/FlashFry/	[35]
CrisFlash	An algorithm-based tool to detect off-target effects incorporates user-supplied variant data with unlimited mismatches.	Alignment	https://github.com/crisflash	[34]
MIT	It can find out potential off-target sites in the early stages of gene editing with 20 bp gRNA without PAM.	Scoring	thttp://www.genome-engineering.org/	[36]
CFD	An extensively used tool for off-target evaluation and detection, with 20 bp gRNA and PAM.	Scoring	http://www.broadinstitute.org/rnai/public/software/index	[37]
CRISTA	Machine learning tool with numerous features, GC contents, RNA secondary structure and epigenetic factors.	Scoring	http://crista.tau.aC.il/	[38]
Elevation	Machine learning with two layers regression model based developed, with epigenetic factors.	Scoring	https://crispr.mL	[39]
DeepCRISPR	The latest deep learning-based tool can predict on-target and off-target cleavage sites simultaneously, with epigenetic factors.	Scoring	http://www.deepcrispr.net/	[40]

**Table 3:** Unbiased methods to detect and evaluate off-target sites in living organisms.

Tools	Description	Advantages	Disadvantages	Cell Lines	Reference
Digenome–seq	Extensively used unbiased off-target in vitro detection method	It can detect off-target sites with 0.1% or lower Indels frequency.	Expensive, require a reference genome, with multiple gRNA sequence depth become challenging.	Various living organism	[46]
SITE–seq	Biochemical unbiased in vitro genome-wide assay to detect off-target of both Cas9 and dCas9 variants	SITE–seq calculate both, mutations and their consequences at the cellular level	–	Human cell lines	[50]
CIRCLE–seq	Highly sensitive, useful unbiased in vitro genome-wide assay, Outperformed than existing in vitro unbiased assays (Digenome–seq and SITE–seq)	Detect SNPs associated off-target effects with a 94% efficiency level at the genomic level.	Sensitivity is low, only work in specific cells tagged by GUIDE–seq dsODN.	Human cell lines	[47]
IDLVs	Unbiased cell-based genome-wide assay to detect off-target effects of all nucleases (CRISPR/Cas9, TALENs and ZFNs)	Effective off-target detecting method in those cells which are difficult to transfect like primary human cells	Less sensitive and cannot detect all off-target effects	Human cell lines	[51]
Chip–seq	Unbiased cell-based genome-wide assay to detect off-target effects of dCas9 nucleases.	More excellent coverage, broad range and direct method to detect off-target effects	Detect only off-target sites associated with dCas9.	Human cell lines	[52]
BLESS	Direct cell-based genome-wide off-target evaluation and detection method	Detect the off-target effects introduced by Cpf1 and Cas9.	It only detects off-target effects during the labeling period and reference genome required.	Mouse and human cell lines	[53]
GUIDE–seq	Off-target detection based on the incorporation of oligodeoxynucleotides (dsODN) in DSBs followed by the NHEJ	Highly sensitive and also can identify translocations	Efficiency limited by chromatin accessibility	Various living organism	[54]
DISCOVER–seq	A dominant, sensitive cell-based genome-wide assay for in vivo off-target detection	Highly sensitive	Detect off-target sites in vivo in adenoviral delivered Cas nucleases	Human and mouse cell lines	[55]
LAM–HTGTS	Robust, sensitive unbiased cell-based genome-wide off-target detection method in CRISPR/Cas9 gene editing	Sensitive and can detect translocations	Limited by chromatin accessibility	Human cell lines	[55]
GOTI	Detect the off-target effects caused by CRISPR/Cas9, Base editors (CBE, ABE)	Detect the off-target sites from derived cells of single blastomeric.	–	Mouse	[56]
VIVO	Latest highly sensitive and effective strategy to detect off-target effects	Used to detect cell-based genome-wide or in vivo off-target effects caused by CRISPR/Cas9 nuclease.	–	Mouse liver cells	[57]

**Table 4:** Characteristics of Cas9 variants and their specificity.

Cas Nucleases	Description	Remarks on Off-Target Effects	Reference
dCas9–FokI	Deactivated SpCas9 fused with the catalytic domain of FokI	Decreased off-target sites and increase on-target activity by 140-fold higher than wild type SpCas9	[85]
SpCas9–HFI	Created through Point mutation in SpCas9	The GUIDE–seq tool used to assess the off-target sites at eight different loci through SpCas9 -HFI. The on-target activity of SpCas9–HFI was higher than WT SpCas9.	[86]
SpCas9 nickase	It engineered through deactivation of the RuvC domain of SpCas9 through mutation	It reduced the off-target effects 1500 times than wild type SpCas9.	[36,65]
Sniper–Cas9	It engineered through mutation in E.coli. The efficiency limited by the large size	The GUIDE–seq performed to evaluate off-target effects. With truncated gRNA and RNP delivery methods, there was no detectable off-target effect.	[87]
eSpCas9	It is engineered through point mutation in SpCas9 at K848A, K1003A and R1060A loci	BLESS analysis used to analyze off-target sites at two different loci. Off-target effects reduced by eSpCas9 than wild type SpCas9.	[88]
evoCas9	Created through point mutation in REC3 domain of SpCas9	GUIDE–seq used to assess the efficiency of evoCas9 at eight different loci. The on-target activity was significantly higher than off-target activity in evoCas9 than wild type SpCas9.	[89]
xCas9	Several point mutations in SpCas9	GUIDE–seq study performed to assess the on-target activity of xCas9 at eight different loci, off-target effects significantly produced by XCas9 were lower than WT SpCas9.	[90]
VRER–SpCas9	It was engineered through point mutation in PAM, recognizing the domain of SpCas9	GUIDE–seq used to evaluate at five different loci targeting by VRER–SpCas9 and SpCas9. The numbers of off-target sites were slightly lower than WT SpCas9.	[91]
Hypa Cas9	Hyper accurate Cas9, created through point mutation in the REC3 domain of SpCas9	GUIDE–seq performed to check on the target activity of Hypa Cas9 at different six loci. Hypa Cas9 induced low off-target effects than SpCas9–HFI and eSpCas9.	[92]
VRQR-SpCas9	It was engineered through point mutation in PAM, recognizing the domain of SpCas9	GUIDE–seq performed to analyze the off-target and on-target activities of VRQR-SpCas9 on eight different loci. The off-target produced by VRQR-SpCas9 is equal to WT SpCas9.	[91]
SaCas9	Nuclease derived from *S. aureus*	GUIDE–seq performed to analyze the off-target and on-target ratios of saCas9 at three different loci. The on-target activity was higher of saCas9 than WT SpCas9.	[93]
St1Cas9	Derived from *Streptococcus* *thermophilus*	Highly specific, but low on-target activity than Cas9.	[94]
St3Cas9	Derived from Streptococcus thermophilus	More significant occurs on-target activity than St1Cas9 and SpCas9.	[95]
NmCas9	Derived from *Neisseria meningitidis*	More specific than wild type SpCas9 in human primary cell lines.	[96]
CjCas9	Smallest Cas9 orthologous, derived from *Campylobacter jejuni*	Reduced off-target sites and on-target activity is higher than SpCas9.	[97]
